# Global Proteomic Analysis of the Resuscitation State of *Vibrio parahaemolyticus* Compared With the Normal and Viable but Non-culturable State

**DOI:** 10.3389/fmicb.2019.01045

**Published:** 2019-05-08

**Authors:** Qingping Zhong, Bin Wang, Jie Wang, Yufei Liu, Xiang Fang, Zhenlin Liao

**Affiliations:** ^1^Guangdong Provincial Key Laboratory of Food Quality and Safety, College of Food Science, South China Agricultural University, Guangzhou, China; ^2^SCAU (Chaozhou) Food Institute Co. Ltd., Chaozhou, China; ^3^Guangdong Scau Assets Management Co., Ltd., South China Agricultural University, Guangzhou, China

**Keywords:** *Vibrio parahaemolyticus*, viable but non-culturable state, resuscitation, proteome, differentially expressed proteins, iTRAQ

## Abstract

*Vibrio parahaemolyticus* is a common pathogen which has become a major concern of seafood products. The bacteria in the viable but non-culturable (VBNC) state are unable to form colonies on growth media, but under appropriate conditions they can regain culturability. In this study, *V. parahaemolyticus* was induced into VBNC state at low temperature and oligotrophic condition, and was resuscitated to culturable state. The aim of this study is to explore the comparative proteomic profiles of the resuscitation state compared with the VBNC state and the exponential phase of *V. parahaemolyticus* using isobaric tags for relative and absolute quantitation (iTRAQ) technique. The differentially expressed proteins (DEPs) were subjected to GO functional annotations and KEGG pathway analysis. The results indicated that a total of 429 proteins were identified as the significant DEPs in the resuscitation cells compared with the VBNC cells, including 330 up-regulated and 99 down-regulated DEPs. Meanwhile, the resuscitation cells displayed 25 up-regulated and 36 down-regulated DEPs (total of 61 DEPs) in comparison with the exponential phase cells. The remarkable DEPs including ribosomal proteins, ABC transporters, outer membrane proteins and flagellar proteins. GO annotation showed that the 429 DEPs were classified into 37 GO terms, of which 17 biological process (BP) terms, 9 cellular component (CC) terms and 11 molecular function (MF) terms. The up-regulated proteins presented in all GO terms except two terms of developmental process and reproduction. The 61 DEPs were assigned to 23 GO terms, the up- and down-regulated DEPs were both mainly involved in cellular process, establishment of localization, metabolic process and so on. KEGG pathway analysis revealed that the 429 DEPs were assigned to 35 KEGG pathways, and the pathways of ribosome, glyoxylate and dicarboxylate metabolism were significantly enriched. Moreover, the 61 DEPs located in 26 KEGG pathways, including the significantly enriched KEGG pathways of ABC transporters and two-component system. This study would contribute to a better understanding of the molecular mechanism underlying the resuscitation of the VBNC state of *V. parahaemolyticus*.

## Introduction

*Vibrio parahaemolyticus* is a gram-negative marine bacterium which can be frequently found in coastal environments and a variety of seafood such as shrimp, prawns, oyster, clam, mussel, crab, and marine fish. It is a significant foodborne pathogen which is usually responsible for food poisoning and acute gastroenteritis (McLaughlin et al., 2005; Nair et al., 2007; Su and Liu, 2007; Yu et al., 2016). In recent years, *V. parahaemolyticus* is becoming the leading foodborne pathogen that leads to food poisoning in China and the United States, and the food poisoning outbreaks caused by *V. parahaemolyticus* have been spread all over the world including Asia, America, Europe, and Africa (Ansaruzzaman et al., 2005; McLaughlin et al., 2005; Drake et al., 2007; Nair et al., 2007; Wu et al., 2014; Xu et al., 2014).

Recently, the viable but non-culturable state (VBNC) of bacteria has attracted great attention and more than 85 species of bacteria have been demonstrated to be capable of entering the VBNC state (Li et al., 2014; Zhao et al., 2017). VBNC bacteria are unable to form colonies on conventional growth media, but are viable and maintain metabolic activity, which may constitute an unrecognized source of food contamination and infection (Oliver, 2005; Li et al., 2014). This state is a survival strategy adopted by some bacteria when they are exposed to adverse environmental conditions, but under favorable conditions they can resuscitate to culturable state and retain pathogenicity (Oliver, 2005, 2010; Bedard et al., 2014). Some studies indicated that the VBNC bacteria in food exhibited virulence after resuscitation in food model systems (Dinu and Bach, 2011; Ferro et al., 2018). Therefore, this state of pathogens has been considered as a significant food safety and public health concern (Li et al., 2014; Ferro et al., 2018).

Although *V. parahaemolyticus* has been reported to enter into VBNC state under harsh environmental stresses (Chen et al., 2009; Lai et al., 2009; Boonyawantang et al., 2012; Su et al., 2013; Yoon et al., 2017), and resuscitate upon exposure to appropriate conditions (Mizunoe et al., 2000; Wong, 2004; Coutard et al., 2007), the responses of the VBNC and resuscitation state of *V. parahaemolyticus* at the proteomic level have not been clarified, especially the protein expressions during the recovery phase remain unclear.

In recent years, a comprehensive protein profiling strategy named as isobaric tags for relative and absolute quantification (iTRAQ) has emerged as a powerful high-throughput proteomics method (Zieske et al., 2005; Wright et al., 2012). As for the iTRAQ-based research on *V. parahaemolyticus*, Yang et al. (2015) presented the proteomic profile of *V. parahaemolyticus* under different culture conditions, but only one iTRAQ-based study on the VBNC state of *V. parahaemolyticus* has been reported previously (Zhong et al., 2018). And there is no global proteomic analysis of the resuscitation state of *V. parahaemolyticus* compared with the normal or VBNC state. In the present study, the iTRAQ method was employed to analyze protein profiles of these three states of *V. parahaemolyticus*. The differentially expressed proteins (DEPs) were identified, and the functional interpretation of the DEPs was carried out. This study would contribute to broadening our understanding on the molecular mechanism underlying the resuscitation of the VBNC state of *V. parahaemolyticus*.

## Materials and Methods

### Bacterial Strain and Culture Conditions

*Vibrio parahaemolyticus* ATCC17802 was obtained from Guangdong Culture Collection Centre of Microbiology (Guangdong, China). The strain was preserved in 10% (w/v) glycerol broth at −80°C and was refreshed twice on tryptic soy agar (TSA) plates containing 3.0% NaCl at 37°C. Then the strain with high activity was cultured into exponential phase in tryptic soy broth (TSB) containing 3.0% NaCl by keeping in a shaker incubator (120 rpm) at 37°C.

### Induction of the VBNC State of *V. parahaemolyticus*

After being cultured overnight, the cells were harvested by centrifugation (Eppendorf, Hamburg, Germany) at 8,000 rpm for 5 min at 4°C, and rinsed twice with 3% NaCl solutions, then the cells were re-suspended in the sterile 3% NaCl at a final density of 10^7^ CFU/mL which was determined by plate counting on 3.0% NaCl-TSA. The cells were kept at 4°C to be induced into the VBNC state. At each designated time points, the culturability and viability of the cells were analyzed, and the VBNC cells were conformed and enumerated as described previously (Liu et al., 2018). All the experiments were carried out in triplicate. The VBNC cells were collected by centrifugation at 10,000 rpm for 5 min for the following experiments.

### Resuscitation of the VBNC Cells

The VBNC cells were collected by centrifugation at 8,000 rpm for 5 min at 4°C, and re-suspended by 9 mL TSB containing 1% Tween 80 (v/v). The cell suspension was incubated at 37°C for 24 h with shaking (120 rpm). Plate counts (on 3% NaCl TSA) were conducted to determine the resuscitation of the VBNC cells.

### Protein Extraction, Digestion and Labeling With iTRAQ Reagents

Protein expression profile analysis was conducted for three samples which were exponential-phase cells (C sample), VBNC cells (V sample) and resuscitation cells (R sample). The cells were washed twice with cooled phosphate-buffered saline (PBS) and were centrifuged at 8,000 rpm for 5 min at 4°C. The collected cells were immediately stored at −80°C. Three biological replicates were used per sample. The bacterial cells were mixed with lysis solution (1% SDS, 200 mM DTT, protease inhibitor cocktail at a volume ratio of 1:100, 50 mM Tris–HCl, pH 8.8) at a ratio of 1:10, and incubated in ice-bath for 30 min, vortex-oscillated for 10 s every 10 min, then incubated at 100°C for 5 min. The mixture was centrifuged at 12,000 g, 4°C for 20 min. The supernatant was added with pre-cooled acetone at a ratio of 1:4. The proteins were precipitated overnight and centrifuged at 12,000 × *g*, 4°C for 20 min. The protein precipitate was mixed with 90% acetone for 10 s and then centrifuged at 12,000 × *g*, 4°C for 20 min. The protein precipitate was dissolved in the protein lysis solution (1% SDS, 8 M urea, protease inhibitor cocktail at a volume ratio of 1:100), and the supernatant was obtained by centrifugation at 4°C for 30 min. The protein concentrations were determined using a BCA Assay Kit (Thermo Fisher Scientific, United States). The sample solution was added with TCEP at the final concentration of 10 mM, and incubated at 37°C for 60 min. Then iodoacetamide was added (40 mM), the reaction took place in the dark at room temperature for 40 min. The solution was mixed with pre-cooled acetone at a volume ratio of 1:6, and put at −20°C for 4 h. The precipitate was collected by centrifugation at 10,000 × *g* for 20 min, dissolved with 100 μL of 100 mM TEAB, and then digested with trypsin at the mass ratio of 25:1 overnight at 37°C. The peptide mixture was labeled with the 8-plex iTRAQ reagent according to the protocol provided by the manufacturer (AB Sciex, United States).

### Chromatographic Separation and MS/MS Analysis

The labeled peptides were resuspended with loading buffer (water, adjusted to pH10 with ammonia and formic acid), separated by high pH reversed-phase liquid chromatography (RPLC) using an Acquity UPLC system (Waters, United States). The gradient elution was performed on C18 column (1.7 μm, 2.1 mm × 150 mm XBridge BEH300, Waters, United States) at a flow rate of 400 μL/min with the gradient increased from 0 to 100% B (B: acetonitrile) in 40 min. Twenty fractions were collected from each sample which were subsequently pooled resulting in ten total fractions per sample. Vacuum centrifugal concentration was performed using rotation vacuum concentration Christ RVC 2–25 (Christ, Germany).

Mass spectrometry analysis was conducted on a Q-Exactive mass spectrometer (Thermo Fisher Scientific, United States) that was coupled with EASY-nLC 1200 (Thermo Fisher Scientific, United States). The peptide mixture was loaded onto the C18-reversed phase column (5 μm, 75 μm × 25 cm, Thermo Fisher Scientific, United States) in 2% B (B: 0.1% formic acid in acetonitrile) and separated with a linear gradient increased from 2 to 80% B in 90 min at a flow rate of 300 nL/min. The Q-Exactive mass spectrometer was operated in the data-dependent mode to switch automatically between MS and MS/MS acquisition. Survey full-scan MS spectra (m/z 350-1300) were acquired with a mass resolution of 70 K, followed by twenty sequential high energy collisional dissociation (HCD) MS/MS scans with a resolution of 17.5 K. In all cases, one microscan was recorded using a dynamic exclusion period of 18 s. For MS/MS, Normalized collision energy was set at 30. Mass spectrometric analyses were performed in duplicate for each biological replicate.

### Proteomic Analysis of the Differential Expression Proteins

The protein sequences of *V. parahaemolyticus* were downloaded from UniProt database^1^. The database searching was performed using Proteome Discoverer Software 2.1. Proteome discoverer database search parameters were showed in Supplementary Table S1. A threshold of 1% false discovery rate (FDR) was used to identify and quantify proteins, and one unique peptide was considered suitable for a reliable protein identification. The differential expression proteins (DEPs) in the resuscitation cells and control samples were identified, the fold change of each protein was calculated based on log_2_ value of the relative abundance between test and control samples. The *p*-value < 0.05 and fold change (FC) >1.50 or <0.67 were used as the threshold to define the significance of protein expression difference. Then DEPs were further subject to functional analysis according to the Gene Ontology (GO)^2^ and KEGG pathway^3^ databases, and were enriched into different functions with *p* ≤ 0.05 as a threshold to determine the significant enrichment of DEPs.

## Results

### Mass Spectrometric Identification of the Proteins, GO and KEGG Pathway Annotations

**FIGURE 1:**
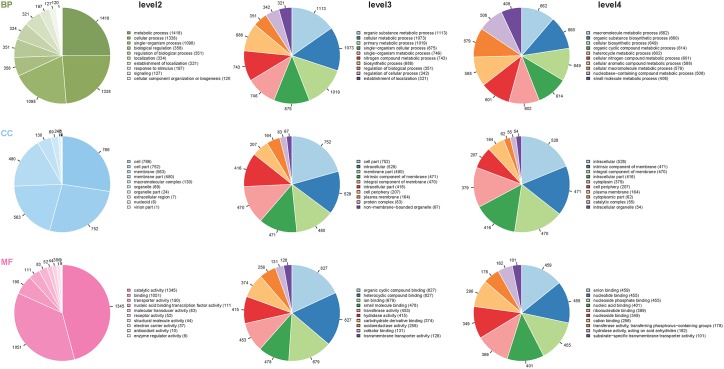
Distribution of the proteins classified into different GO terms at the second, third and fourth levels. The different colors in pies represent different GO terms. The area is the relative proportion of proteins in each GO term. The nine pie charts were sorted by the three main categories, BP, biological processes; CC, cellular components, and MF, molecular function from up to down and level of second, third and forth from left to right. The descriptions of BP, CC, and MF are more detailed with the increase of level numbers.

**FIGURE 2:**
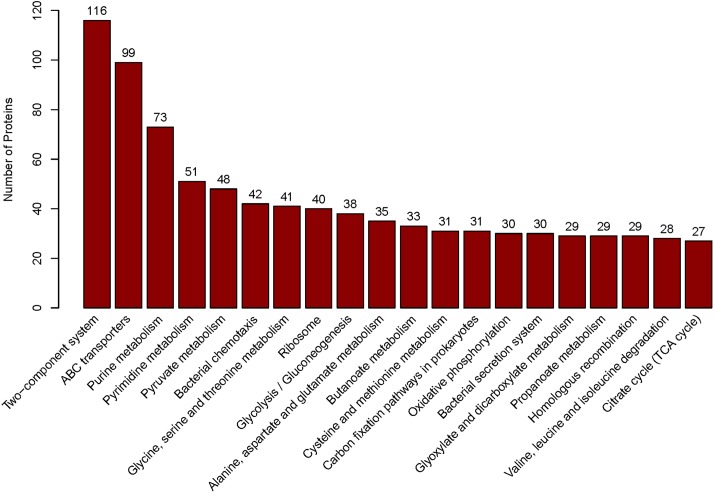
The top 20 pathways. Those pathways were sorted by the protein numbers which annotated the corresponding pathway. Therefore, more annotated proteins involved in the pathway, the higher bar is.

We applied ITRAQ technique to analyze comparative proteomic profiles, the total spectrum, identified spectrum, peptide number, protein number were showed in Supplementary Table S1. All the identified proteins were presented in detail in Supplementary Table S2, and were subjected to GO and KEGG annotations to understand the biological functions. The GO database contains three ontologies: biological process (BP), cellular component (CC), and molecular function (MF). A total of 2625 identified proteins were categorized into the three main categories, at 39 secondary levels, 174 third levels and 476 forth levels of GO terms. At secondary level, metabolic process, cell and catalytic activity were the most significant GO terms in BP, CC and MF, respectively. At level 4, macromolecule metabolic process, intracellular and anion binding were the most significant GO terms in BP, CC and MF, respectively (Figure 1). In addition, the top 20 pathways with the largest number of the identified proteins were presented in Figure 2. The pathways of two-component system, ABC transporters and purine metabolism had 116, 99 and 73 proteins, respectively.

### Differentially Expressed Proteins (DEPs)

As the fold change >1.50 or <0.67 and *p*-value < 0.05 were used as the threshold to define the significance of protein expression difference, 429 proteins were identified as the significant DEPs in comparison with the resuscitation cells and the VBNC cells. Scatter plots were generated to visualize the distribution of expression variation of proteins (Supplementary Figure S1). Of the 429 DEPs, 330 were significantly up-regulated and 99 were down-regulated. Some DEPs were showed in Tables 1, 2. Among the remarkably up-regulated proteins, the ribosomal proteins such as 30S ribosomal protein S19 OS (A6B9I5), 50S ribosomal protein L24 OS (A6B9H4), 50S ribosomal protein L17 OS (A6B9B5) and ribosomal protein S7 OS (A6BBF2) showed notable up regulation. Moreover, integration host factor subunit alpha OS (A6B883), twin-arginine translocation pathway signal OS (A6B1K7), and thioredoxin OS (A6B646) were over-expressed significantly (Table 1). In addition, some ABC transporters were significantly up-regulated, of which phosphate ABC transporter, periplasmic phosphate-binding protein OS (A6AX32); ABC transporter, periplasmic substrate-binding protein OS (A6B7T6) and ABC transporter substrate-binding protein (A6B1I7) expressed at much higher level, with the fold change of 6.1247, 2.8945, and 2.5739, respectively (Table 1 and Supplementary Table S3A). The results revealed a significant up regulation of some important proteins in the resuscitation state, these could result in the increase of protein synthesis and some physiological activity. Of the down-regulated proteins (Table 2), TrkA-N domain protein OS (A6AX62), PhoH family protein OS (A6B7I9), outer membrane lipoprotein OS (A6BA68) and potassium uptake protein (A6AX63) were expressed at lower level in the resuscitation cells than in the VBNC cells. The detailed information of all the DEPs was listed in Supplementary Table S3A.

**Table 1:** Some significantly up-regulated proteins in the resuscitation cells compared with VBNC cells of *V. parahaemolyticus*.

Accession	Description	FC	log2FC	GO	KO	COG
A6B9I5	30S ribosomal protein S19 OS	6.575916	2.717192	GO:0003723; GO:0003735; GO:0005840; GO:0006412; GO:0015935; GO:0019843; GO:0030529	K02965	COG0185
A6AX32	Phosphate ABC transporter, periplasmic phosphate-binding protein OS	6.124724	2.614645	GO:0006810	K02040	COG0226
A6B9H4	50S ribosomal protein L24 OS	4.952381	2.308122	GO:0003723; GO:0003735; GO:0005622; GO:0005840; GO:0006412; GO:0019843; GO:0030529	K02895	COG0198
A6B9B5	50S ribosomal protein L17 OS	4.873684	2.285013	GO:0003735; GO:0005622; GO:0005840; GO:0006412; GO:0030529	K02879	COG0203
A6B883	Integration host factor subunit alpha OS	4.5672269	2.191318	GO:0003677; GO:0006310; GO:0006351; GO:0006355; GO:0006417	K04764	COG0776
A6B1K7	Twin-arginine translocation pathway signal OS	4.505689	2.171748	–	K07093	COG3211
A6BBF2	Ribosomal protein S7 (Fragment) OS	4.129496	2.045966	GO:0003723; GO:0003735; GO:0005840; GO:0006412; GO:0015935; GO:0019843; GO:0030529	K02992	COG0049
A6B646	Thioredoxin OS	3.590717	1.844272	GO:0005623; GO:0006662; GO:0015035; GO:0045454; GO:0055114	K03671	COG0526
A6BAY8	50S ribosomal protein L28 OS	3.504983	1.809408	GO:0003735; GO:0005622; GO:0005840; GO:0006412; GO:0030529	K02902	COG0227
A6B6K0	30S ribosomal protein S21 OS	3.487455	1.802175	GO:0003735; GO:0005622; GO:0005840; GO:0006412; GO:0030529	K02970	COG0828
A6B3T6	30S ribosomal protein S15 OS	3.479876	1.799036	GO:0003723; GO:0003735; GO:0005622; GO:0005840; GO:0006412; GO:0019843; GO:0030529	K02956	COG0184
A6B0V5	Proton/glutamate symporter OS	3.432071	1.77908	GO:0015293; GO:0016020; GO:0016021; GO:0055085	–	COG1301
A6B5K1	DNA-binding protein HU-beta OS	3.327703	1.734527	GO:0003677; GO:0030261	K05787	COG0776
A6AX92	Periplasmic maltose-binding protein OS	3.258359	1.704145	GO:0005215; GO:0005363; GO:0006810; GO:0015768	K10108	COG2182
A6B3L4	Pirin domain protein OS	3.127946	1.645216	–	K06911	COG1741
A6B3R6	DNA-binding protein OS	3.098160	1.631411	GO:0003677; GO:0005622; GO:0006355; GO:0046983	K03746	COG2916
A6B613	Polar flagellar FlgM OS	3.090032	1.6276218	GO:0045892	K02398	COG2747

**Table 2:** Some significantly down-regulated proteins in the resuscitation cells compared with VBNC cells of *V. parahaemolyticus.*

Accession	Description	FC	log2FC	GO	KO	COG
A6B843	Uncharacterized protein OS	0.251340	−1.99229	GO:0016020; GO:0016021	–	–
A6B5A3	Uncharacterized protein OS	0.332894	−1.58687	–	–	–
A6AX62	TrkA-N domain protein OS	0.355519	−1.492	GO:0006813; GO:0008324; GO:0098655	K03499	COG0569
A6B7I9	PhoH family protein OS	0.398355	−1.32787	GO:0005524	K07175	COG1875
A6B969	Uncharacterized protein OS	0.425146	−1.23397	GO:0008152; GO:0016491; GO:0055114	–	COG4230
A6BA68	Outer membrane lipoprotein OS	0.454938	−1.13626	GO:0019867	K06078	COG4238
A6AX63	Potassium uptake protein, TrkH family OS	0.462428	−1.1127	GO:0006812; GO:0008324; GO:0016020; GO:0016021; GO:0022820; GO:0055085; GO:0071805	K03498	COG0168
A6B4E2	ATP synthase C chain OS	0.473320	−1.07911	GO:0006810; GO:0006811; GO:0015078; GO:0015986; GO:0015991; GO:0015992; GO:0016020; GO:0016021; GO:0016787; GO:0033177; GO:0045263	K02110	COG0636
A6B2D1	Isochorismatase family protein OS	0.487431	−1.03673	GO:0003824; GO:0008152	–	COG1335

Compared with the exponential phase cells, the resuscitation cells displayed 61 significant DEPs, including 25 up-regulated and 36 down-regulated DEPs. Some notably DEPs were indicated in Tables 3, 4. A few proteins presented at increased abundance in the resuscitation cells, such as phosphate ABC transporter (A6AX32), twin-arginine translocation pathway signal OS (A6B1K7), outer membrane protein OS (A6B195). The proteins displayed down-regulated were putative outer membrane porin protein locus of qsr prophage OS (A6B101), acetyl-CoA carboxylase, biotin carboxyl carrier protein OS (A6B7L0), ABC-type proline/glycine betaine transport system, periplasmic component OS (A6AZ90), and so on. The Supplementary Table S3B presented the detailed information of these DEPs.

**Table 3:** Some significantly up-regulated proteins in the resuscitation cells compared with exponential-phase cells of *V. parahaemolyticus*.

Accession	Description	FC	log2FC	GO	KO	COG
A6AX32	Phosphate ABC transporter, periplasmic phosphate-binding protein OS	5.549	2.472228	GO:0006810	K02040	COG0226
A6B4C7	Uncharacterized protein OS	4.118	2.041944	–	–	–
A6B1K7	Twin-arginine translocation pathway signal OS	3.564	1.833497	–	K07093	COG3211
A6B195	Outer membrane protein OS	2.205	1.140779	–	–	COG3203
A6B4Y1	Alkaline phosphatase OS	1.912	0.935082	GO:0003824; GO:0008152; GO:0016311; GO:0016791	K01077	COG1785
A6BCI2	P pilus assembly/Cpx signaling pathway, periplasmic inhibitor/zinc-resistance associated protein (Fragment) OS	1.904	0.929033	GO:0042597	K06006	COG3678
A6B7T6	ABC transporter, periplasmic substrate-binding protein OS	1.893	0.920674	GO:0006810	K02040	COG0226

**Table 4:** Some significantly down-regulated proteins in the resuscitation cells compared with exponential-phase cells of *V. parahaemolyticus*.

Accession	Description	FC	log2FC	GO	KO	COG
A6B101	Putative outer membrane porin protein locus of qsr prophage OS	0.221	−2.17788	–	–	–
A6B7L0	Acetyl-CoA carboxylase, biotin carboxyl carrier protein OS	0.386	−1.37333	GO:0003989; GO:0006633; GO:0009317	K02160	COG0511
A6AZ90	ABC-type proline/glycine betaine transport system, periplasmic component OS	0.403	−1.31115	GO:0005215; GO:0006810; GO:0015871; GO:0033265; GO:0042597	K02002	COG2113
A6B8M3	Transport-associated OS	0.405	−1.30401	–	–	–
A6AZ88	NAD/NADP-dependent betaine aldehyde dehydrogenase OS	0.429	−1.22095	GO:0008152; GO:0008802; GO:0016491; GO:0016620; GO:0019285; GO:0046872; GO:0055114	K00130	COG1012
A6AZ92	Glycine betaine transport ATP-binding protein opuAA OS	0.432	−1.2109	GO:0000166; GO:0005524; GO:0015220; GO:0015418; GO:0015871; GO:0016787; GO:0016887; GO:0055052; GO:0055085	K02000	COG4175
A6AZ89	Oxygen-dependent choline dehydrogenase OS	0.452	−1.14561	GO:0008812; GO:0016491; GO:0016614; GO:0019285; GO:0050660; GO:0055114	K00108	COG2303
A6B3M1	Sodium/glutamate symporter OS	0.457	−1.12973	GO:0015501; GO:0015813; GO:0016020; GO:0016021; GO:0089711	K03312	COG0786

### GO Annotation and GO Enrichment of the Significant DEPs

**FIGURE 3:**
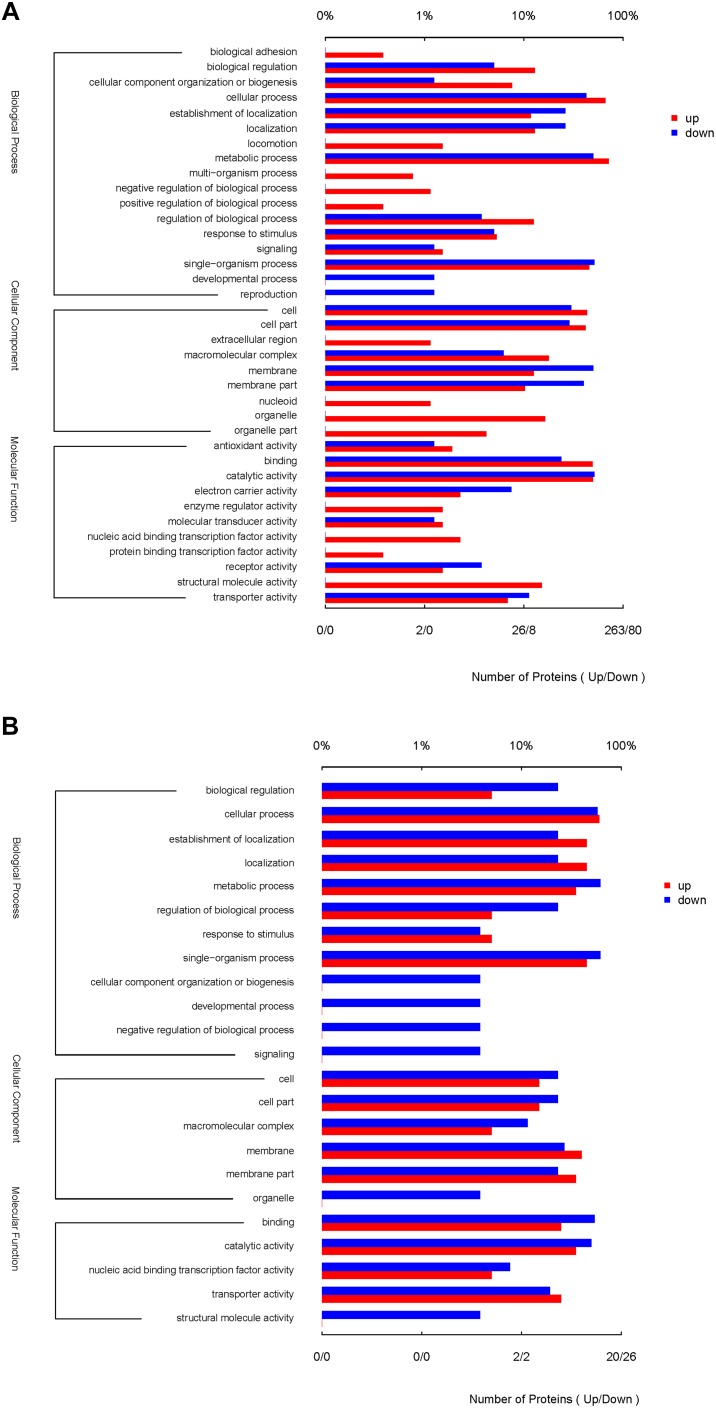
GO terms of the significantly DEPs of the resuscitation cells compared with the VBNC **(A)** or exponential-phase **(B)** cells of *V. parahaemolyticus.* The red bars represented the up-regulated proteins, and blue bars for down-regulated proteins.

The functional interpretation of the significant DEPs was carried out by GO annotation. During the reversion from the VBNC to the culturable state, 429 proteins were differentially expressed and classified into 37 GO terms (Figure 3A), of which 17 BP terms, 9 CC terms and 11 MF terms. The up-graduated proteins involved in all terms except two terms of developmental process and reproduction. In the BP ontology, the DEPs primarily associated with metabolic process (containing 189 up- and 40 down-regulated proteins), cellular process (174 up- and 34 down-regulated proteins), and single-organism process (120 up- and 41 down-regulated proteins). For the CC ontology, the DEPs located mainly in cell, cell part, membrane and organelle. According to GO annotation of MF, the DFPs were involved in catalytic activity (131 up- and 41 down-regulated proteins), binding (129 up- and 19 down-regulated proteins), and so on. In addition, only up-regulated proteins were associated with the 13 GO functional groups, including organelle, structural molecule activity, organelle part, and nucleic acid binding transcription factor activity, with 43, 40, 11, and 6 up-regulated proteins, respectively.

Compared with the exponential-phase cells, the 61 DEPs of the resuscitation cells were grouped into 23 GO terms (Figure 3B). The up- and down-graduated DEPs were both mainly involved in cellular process, establishment of localization, metabolic process, single-organism process, membrane, catalytic activity and so on. However, only one down-graduated DEP was associated with developmental process, negative regulation of biological process, signaling, cellular component organization or biogenesis, and structural molecule activity, respectively. The results indicated that the resuscitation cells recovered to high extent from the VBNC state.

**FIGURE 4:**
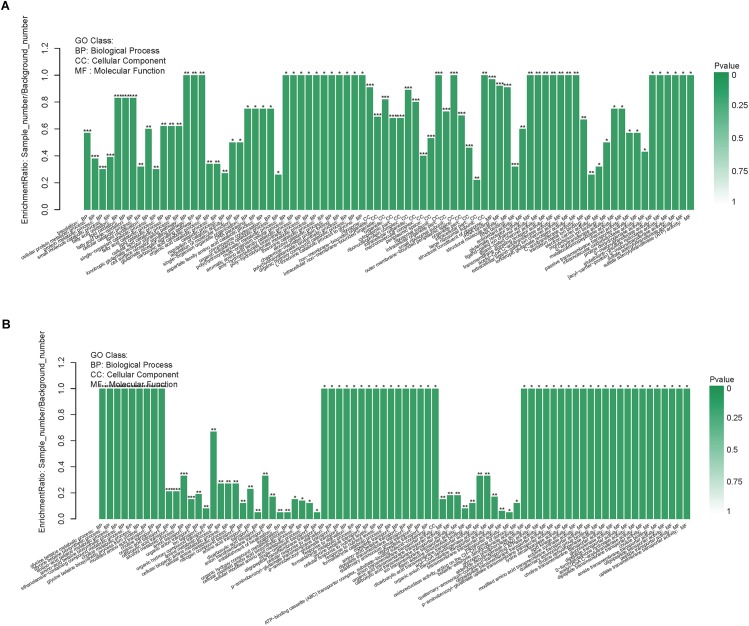
GO enrichment of DEPs in the resuscitation cells compared with the VBNC cells **(A)** or the exponential-phase cells **(B)**. Each bar represented one GO term, which was marked ^∗∗∗^*p* < 0.001, ^∗∗^*p* < 0.01, and ^∗^*p* < 0.05.

The GO enrichment analysis of the DEPs could present the functional enrichment of the DEPs and clarify the difference of samples at the functional level. Figure 4A displayed GO enrichment results of the DEPs in the resuscitation cells versus VBNC cells. Many GO terms were enriched dramatically, for example in the BP ontology, ionotropic glutamate receptor signaling pathway, cell surface receptor signaling pathway, glutamate receptor signaling pathway, fatty acid oxidation, lipid oxidation and fatty acid beta-oxidation were notably enriched. For the CC ontology, many GO terms such as ribosome, intracellular non-membrane-bounded organelle, ribonucleoprotein complex, ribosomal subunit and small ribosomal subunit were enriched significantly. Moreover, the molecular functions such as rRNA binding, structural molecule activity, glutamate receptor activity, ligand-gated ion channel activity, transmembrane signaling receptor activity and acetyl-CoA C-acyltransferase activity were enriched dramatically. More detailed GO enrichment information of the DEPs was listed in Supplementary Table S3C.

In comparison with the exponential-phase cells, the GO enrichment analysis of the DEPs of the resuscitation cells were also conducted. The results showed that the significantly enriched GO terms were belong to BP and MF, such as glycine betaine metabolic process, glycine betaine biosynthetic process, amino-acid betaine metabolic process, choline metabolic process, modified amino acid transport, glycogen phosphorylase activity, aldehyde dehydrogenase (NAD) activity, oligopeptide transmembrane transporter activity, lysine decarboxylase activity and lipid kinase activity (Figure 4B and Supplementary Table S3D).

### KEGG Pathway Analysis of the Significant DEPs

**FIGURE 5:**
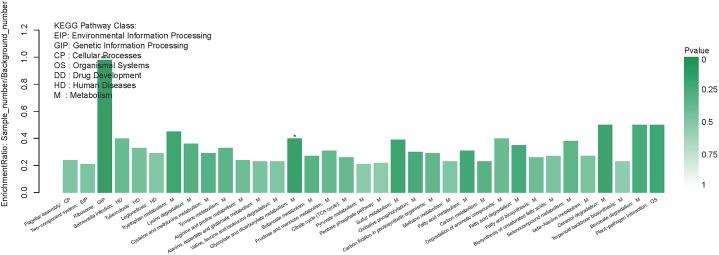
KEGG pathway enrichment of DEPs in the resuscitation cells in comparison with the VBNC cells. Each bar represented one KEGG pathway. Pathway was marked ^∗∗∗^*p* < 0.001 and ^∗^*p* < 0.05. Color of the bars was related to the *p*-value. Decreasing of the *p*-value was corresponded to the color from light to dark.

**FIGURE 6:**
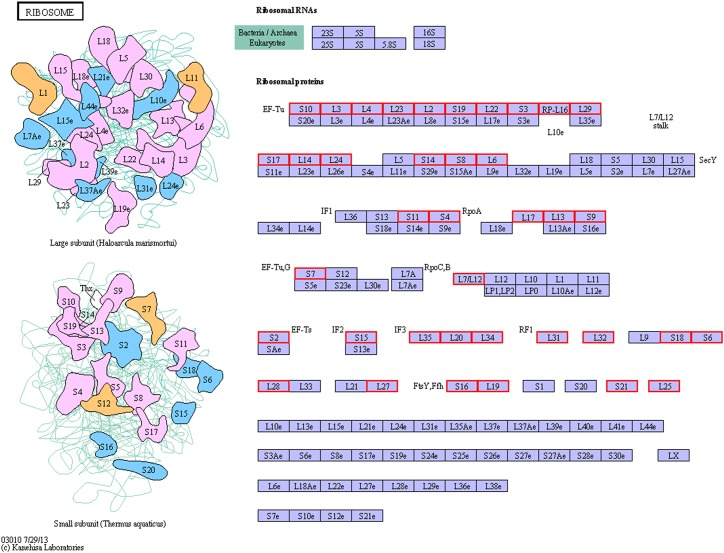
KEGG pathway map of ribosome of the resuscitation cells compared with the VBNC cells. Proteins in blue block belonged to the experimental species. Up-regulated proteins were marked by red frames while down-regulated proteins were marked by green frames.

In addition to the functional GO annotation, KEGG pathway analysis were also conducted on the significant DEPs, in order to provide a comprehensive understanding on the protein profiles of the resuscitation cells of *V. parahaemolyticus*. After conversion from the VBNC to resuscitation state, 429 DEPs were found and assigned to 35 KEGG pathways (Figure 5), and a majority of the pathways related to metabolism (M), 3 pathways associated with human diseases (HD), 1 pathway for environmental information processing (EIP), 1 for genetic information processing (GIP). The KEGG pathways of ribosome, glyoxylate and dicarboxylate metabolism were significantly enriched. Ribosome was the most significant pathway with 39 up-regulated DEPs (Figure 6).

**FIGURE 7:**
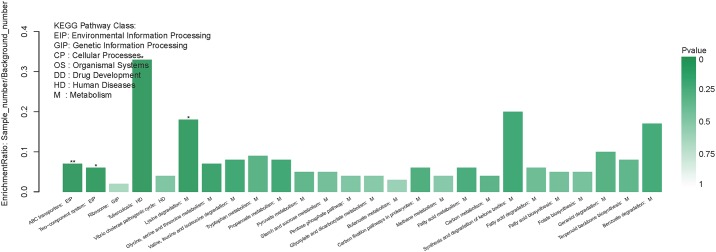
KEGG pathway enrichment of DEPs in the resuscitation cells compared with the exponential-phase cells. Each bar represented one KEGG pathway. Pathway was marked ^∗∗^*p* < 0.01 and ^∗^*p* < 0.05. Color of the bars was related to the *p*-value. Decreasing of the *p*-value was corresponded to the color from light to dark.

**FIGURE 8:**
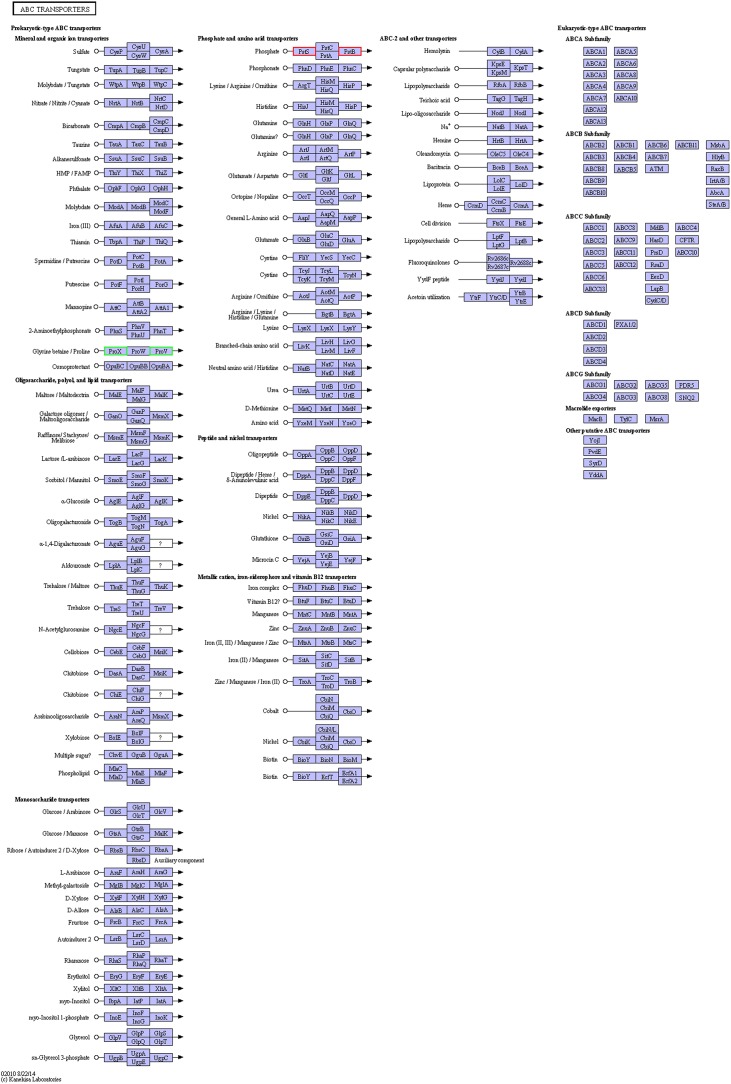
KEGG pathway map of ABC transporters of the resuscitation cells compared with the exponential-phase cells. Proteins in blue block belonged to the experimental species. Up-regulated proteins were marked by red frames while down-regulated proteins were marked by green frames.

Compared with the exponential-phase cells, the DEPs of the resuscitation cells were assigned to 26 KEGG pathways, including 21 metabolism pathways (M), 2 human diseases pathways (HD), 2 environmental information processing (EIP) pathways, and 1 genetic information processing (GIP). The significantly enriched KEGG pathways including ABC transporters, and two-component system (Figure 7). ABC transporters was the most significant pathway with 2 up-regulated and 2 down-regulated DEPs (Figure 8).

## Discussion

Up to now, a number of studies have carried out on *V. parahaemolyticus* to reveal the behavior, entry into the VBNC state in response to various environmental conditions and resuscitation to culturable state when exposed to favorable conditions, and characterize the biochemical properties of the VBNC state (Mizunoe et al., 2000; Wong, 2004; Coutard et al., 2007; Chen et al., 2009; Boonyawantang et al., 2012; Su et al., 2013; Yoon et al., 2017), as well as develop rapid detection methods (Zhong et al., 2016; Liu et al., 2018). However, little studies have reported to analyze the gene or protein expression profiles (Lai et al., 2009; Meng et al., 2015; Zhong et al., 2018). To our knowledge, there is no information concerning the comprehensive protein expression profile of the resuscitation state of *V. parahaemolyticus.*

In our study, the global comparative proteomic profiles of the resuscitation cells compared with the VBNC state and the exponential phase of *V. parahaemolyticus* were studied by iTRAQ technique. In the VBNC state, the levels of the proteins related to ribosome, rRNA binding and tRNA binding were reduced, and the transcription and translation were generally minimized (Zhong et al., 2018). In the present study, after transition from the VBNC to resuscitation state, almost all ribosomal proteins (39/40) were over-expressed notably (Supplementary Table S3A). Among them, some ribosomal proteins were the most distinguished DEPs (Table 1). These 30S and 50S ribosomal subunit proteins constitute ribosome and exhibit different functions in protein synthesis (Yang et al., 2015). Furthermore, GO terms related to ribosome were enriched remarkably (Figure 4). The KEGG pathway analysis also indicated that the pathway of “Ribosome” was the most significantly enriched pathway (Figure 5). This implicating the expression and synthesis of proteins were extensively enhanced to meet a greater requirement of cell growth.

The membrane proteins of bacteria play a crucial role during many cellular and physiological processes, involving in antibiotic efflux system, secretion system, transport, etc., ATP-binding cassette (ABC) transporters are multidomain integral membrane proteins that utilize the energy of ATP hydrolysis to translocate solutes across cellular membranes, and they form one of the largest of all protein families. Through the transport of molecules such as ions, sugars, amino acids, vitamins, peptides, polysaccharides, hormones, lipids and xenobiotics, ABC transporters play major roles in diverse cellular processes such as maintenance of osmotic homeostasis, nutrient uptake, resistance to xenotoxins, antigen processing, cell division, pathogenesis, cholesterol and lipid trafficking (Jones and George, 2004; Rees et al., 2009). In the present study, more than ten ABC transporters expressed at higher level in the resuscitation cells compared with the VBNC cells, and it is worth noting that the 3 top up-regulated ABC transporters (A6AX32, A6B7T6, and A6B1I7) also showed increased abundance compared with the normal cells (Supplementary Tables S3A,B). This suggested that the resuscitated bacterial cells accelerated the synthesis of ABC transporters and increased the *trans*-membrane transport function and the intra- and extra-cellular substance exchange, thereby promoting nutrient absorption and excretion of metabolic products. These key DEPs might be the potential regulatory proteins which deserve much more attention and further study.

In addition, we also observed that the resuscitation cells exhibited higher abundance of Type I secretion target GGXGXDXXX repeat (2 copies) domain protein OS (A6B7Z2) than the VBNC cells and normal cells, with the fold change of 2.252 and 1.732. Investigation of secretion systems is critical to understanding the virulence mechanisms of pathogens. The results also showed that cation/multidrug efflux pump OS (A6B3Y5) expressed at increased level (fold change 1.618) in the resuscitation cells compared with the VBNC cells. Drug efflux pumps can participate in drug resistance to multiple antimicrobials through export drugs, and play other roles in bacteria (Yang et al., 2015). As a result, the VBNC cells may recover the pathogenicity and drug resistance after resuscitation which deserves much more attention.

The outer membrane proteins (OMPs) play key roles in adaptation to changes of external environments due to their location at the outermost area of the cell (Xu et al., 2005). The stressed environmental conditions may pose significant expression changes of OMPs during the VBNC state of bacteria (Xu et al., 2005; Abdallah et al., 2009, 2010; Parada et al., 2016; Zhong et al., 2018). Abdallah et al. (2010) indicated that *V. parahaemolyticus* modified the expression of certain OMPs (e.g., OmpW, OmpA, maltoporin) to respond to gamma irradiation exposure. In the present study, several OMPs were detected at lower abundance in the resuscitated cells, such as outer membrane protein OS (A6BA68); outer membrane porin, OprD family OS (A6B423); putative outer membrane protein, Ail and OmpX OS (A6B7X2); outer membrane protein OmpA OS (A6B0M6), with the fold change of 0.4549, 0.5506, 0.5679, and 0.5878, respectively. These DEPs perhaps were key players or regulators in the VBNC state, but after resuscitation their expression were down-regulated.

The flagellum is a key feature affecting the morphology, chemotaxis, behavior and survival of bacteria (Chilcott and Hughes, 2000). Movement is required for pathogenicity of *V. parahaemolyticus* (Perez-Acosta et al., 2018). Our previous study indicated that the proteins associated with the flagellum were remarkably down-regulated in the VBNC state, the motility decreased and the cells congregated in close proximity (Zhong et al., 2018). In the present study, 5 flagellar proteins were significantly up-regulated in the resuscitation cells, including polar flagellar FlgM OS (A6B613), polar flagellar FlgN OS (A6B614), and flagellin OS (A6B026). In addition, P pilus assembly/Cpx signaling pathway, periplasmic inhibitor/zinc-resistance associated protein (A6BCI2) displayed higher expression in the resuscitation cells than in the VBNC cells and normal cells. GO annotation results also demonstrated that the up-regulated DEPs were involved in the biological processes of locomotion and biological adhesion (Figure 2). Pilus contributes to the adhesion and is important for colonization of bacteria. Furthermore, up-regulating of flagellar proteins and adhesins improved the virulence of *V. parahaemolyticus* (Perez-Acosta et al., 2018). These notably up-regulated proteins were suggested to be the key elements which contribute to the normal viability and function of *V. parahaemolyticus.*

Based on the analysis of GO annotations and KEGG pathways, we found that the DEPs in the resuscitation cells were comprehensively up-regulated, which associated with cellular process, metabolic process, single-organism process, localization, membrane, catalytic activity, transporter activity, binding, and so on. Many GO terms were enriched dramatically, for example ionotropic glutamate receptor signaling pathway, cell surface receptor signaling pathway, fatty acid oxidation, rRNA binding, structural molecule activity, and transmembrane signaling receptor activity. Moreover, compared with normal cells, main up-regulated DEPs were related to cellular process, localization and membrane. The results indicated that the resuscitation cells recovered to high extent from the VBNC state. Furthermore, a majority of the KEGG pathways were related to metabolism. The pathways of ribosome and ABC transporters were the most significantly enriched pathways compared with the VBNC cells and the exponential-phase cells, respectively. These KEGG pathways were presented in Figure 6, 8, 9. These results confirmed that the resuscitated cells of *V. parahaemolyticus* increased the expression and synthesis of proteins extensively to support the cell functions and growth, and some proteins involved in ABC transporters, pilus, type I secretion system, etc., were over-expressed in comparison with the exponential-phase cells.

**FIGURE 9:**
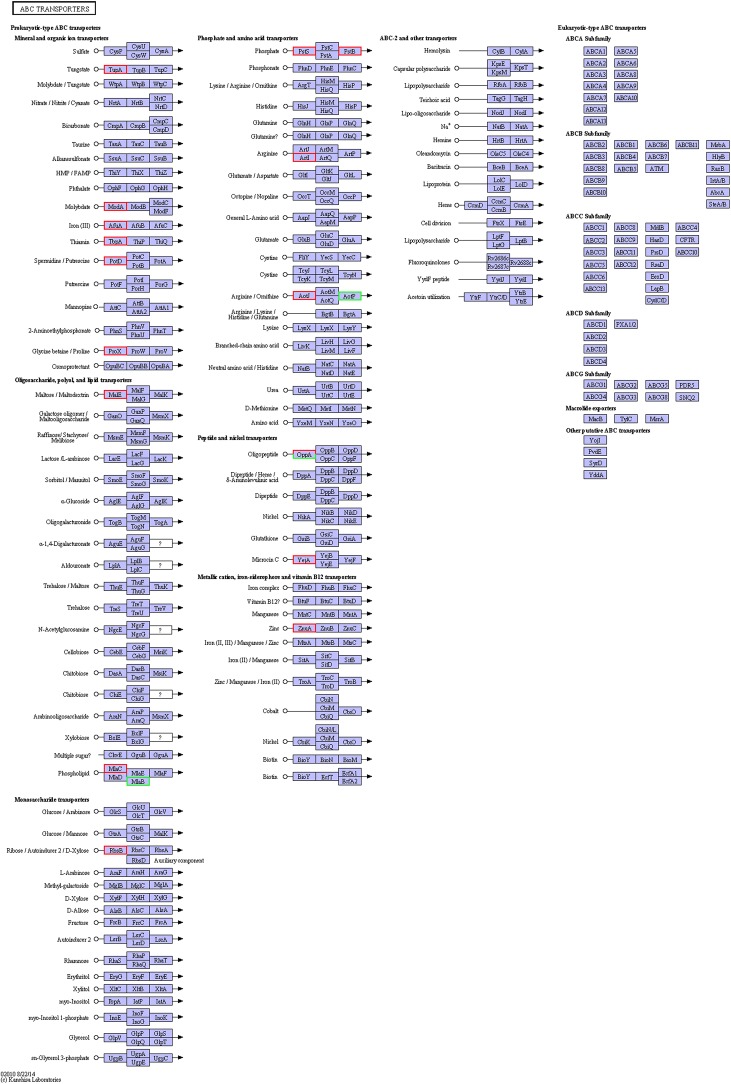
KEGG pathway map of ABC transporters of the resuscitation cells compared with the VBNC cells. Proteins in blue block belonged to the experimental species. Up-regulated proteins were marked by red frames while down-regulated proteins were marked by green frames.

## Conclusion

In conclusion, this study was carried out to explore the comparative proteomic profiles of the resuscitation state compared with the VBNC state and the exponential phase of *V. parahaemolyticus* using iTRAQ method. The results indicated that the DEPs in the resuscitation cells were comprehensively up-regulated, which involved in protein synthesis, *trans*-membrane transport, secretion system, movement, adhesion and other vital processes. The pathways of ribosome and ABC transporters were the most significantly enriched pathways. The remarkable DEPs such as ABC transporters, ribosomal proteins and flagellar proteins might be the potential regulatory proteins or biomarkers associated with the responses of *V. parahaemolyticus* during resuscitation from the VBNC state, and this study would help broaden our understanding of the mechanisms underlying the VBNC and resuscitation states of *V. parahaemolyticus.*

## Author Contributions

QZ analyzed the data and prepared the manuscript. JW contributed to manuscript discussion. BW and YL designed and performed the experiments. XF and ZL contributed to manuscript revision.

## Conflict of Interest Statement

The authors declare that the research was conducted in the absence of any commercial or financial relationships that could be construed as a potential conflict of interest.

## Footnotes

1 http://www.uniprot.org/proteomes/UP000004323

2 http://www.geneontology.org/

3 http://www.genome.jp/kegg/

## Supplementary Material

FIGURE S1 Scatter plot for DEPs of the resuscitation cells compared with the VBNC **(A)** or exponential-phase cells **(B)**. Each point represents a particular protein. Red dots mean up-regulate proteins while green dots mean down-regulate proteins and gray ones for no significant differential expression proteins.

TABLE S1 Proteome discoverer database search parameters.

TABLE S2 Detail information of the identified proteins.

TABLE S3 The DEPs and the GO enrichment information. **(A)** The DEPs of the resuscitation cells compared with the VBNC cells. **(B)** The DEPs of the resuscitation cells in comparison with the exponential-phase cells. **(C)** GO enrichment of the DEPs of the resuscitation cells compared with the VBNC cells. **(D)** GO enrichment of the DEPs of the resuscitation cells compared with the exponential-phase cells.
