# Supplementary material for: Global Proteomic Analysis of the Resuscitation State of Vibrio parahaemolyticus Compared With the Normal and Viable but Non-culturable State
Source: Front Microbiol. 2019 May 8;10:1045. doi: 10.3389/fmicb.2019.01045 (PMC6517545; doi:10.3389/fmicb.2019.01045)
Supplement: FIGURE S1 — Scatter plot for DEPs of the resuscitation cells compared with the VBNC (A) or exponential-phase cells (B). Each point represents a particular protein. Red dots mean up-regulate proteins while green dots mean down-regulate proteins and gray ones for no significant differential expression proteins. [file Data_Sheet_1.docx]

A


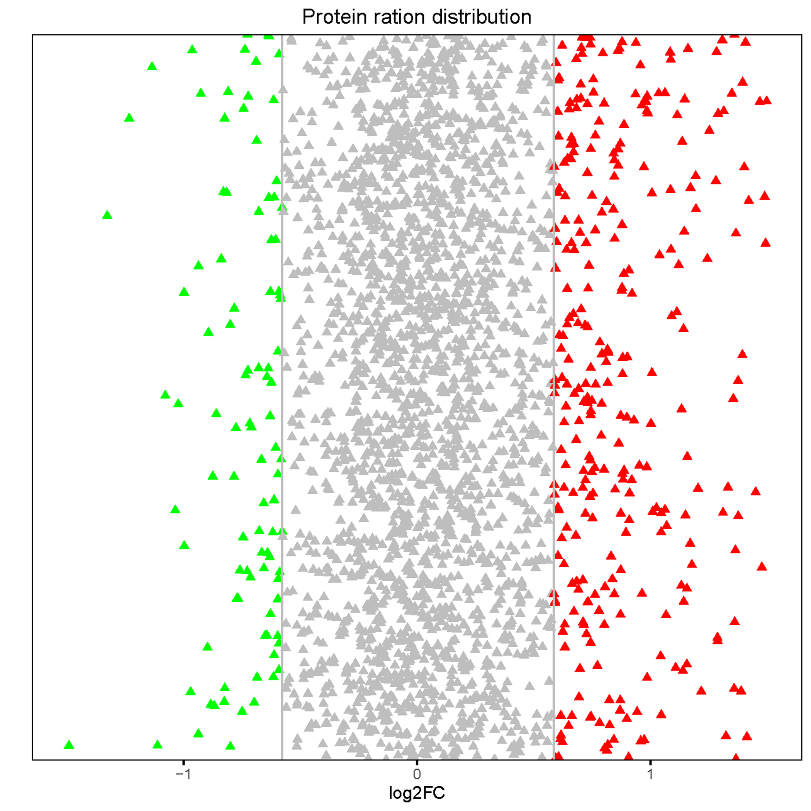


B


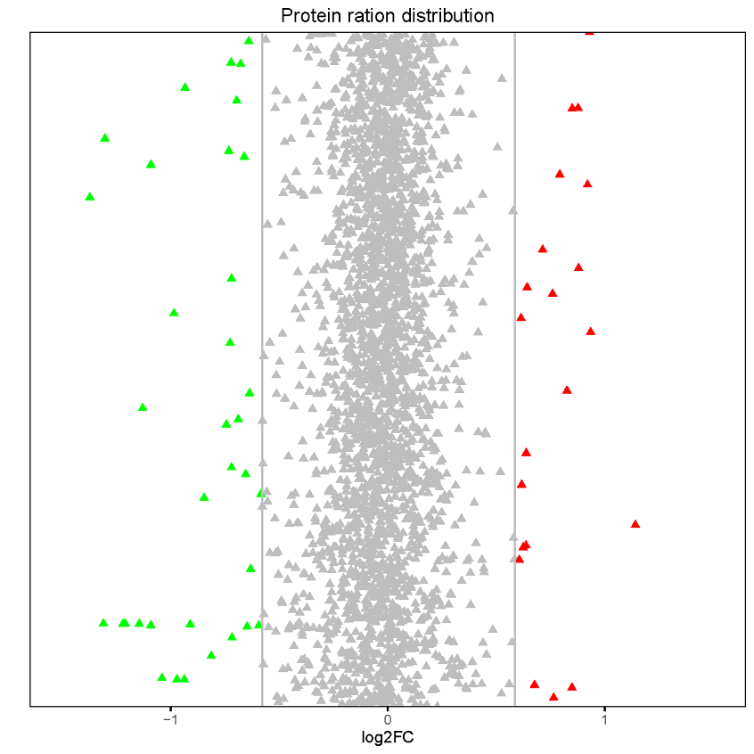


Figure S1 Scatter plot for DEPs of the resuscitation cells compared with the VBNC (A) or exponential-phase cells (B). Each point represents a particular protein. Red dots mean up-regulate proteins while green dots mean down-regulate proteins and grey ones for no significant differential expression proteins.
