# Supplementary material for: Global Proteomic Analysis of the Resuscitation State of Vibrio parahaemolyticus Compared With the Normal and Viable but Non-culturable State
Source: Front Microbiol. 2019 May 8;10:1045. doi: 10.3389/fmicb.2019.01045 (PMC6517545; doi:10.3389/fmicb.2019.01045)
Supplement: TABLE S1 — Proteome discoverer database search parameters. [file Table_1.docx]

Supplementary file 1

Table S1 Proteome discoverer database search parameters

| Item | Value |
| --- | --- |
| ProteomeDiscvererversion: | 2.1 |
| Protein database | uniprot-proteome-Vibrio Parahaemolyticus-5410s-20160721.fasta |
| Cys alkylation | Carbamidomethyl |
| Enzyme name | Trypsin (Full) |
| Max. missed cleavage sites | 2 |
| Precursor mass tolorance | 10 ppm |
| Fragment mass Tolorance | 0.05Da |

Note: Peptide FDR ≤0.01
